# Notes on Preparations for the Sick

**Published:** 1894-09

**Authors:** 


### Notes on Preparations for the Sick.

**Expectorant Pastilles. Voice Tabellæ.**—ALLEN & HANBURYS, London.—The pastilles contain gr. $\frac{1}{40}$ of morphia, gr. $\frac{1}{5}$ of ipecacuanha, and gr. $\frac{1}{5}$ of squill. The tabellæ contain of chlorate of potash gr. iv., borax gr. j., and cocaine gr. $\frac{1}{20}$. Their uses are too well known to need any special reference.

**Tabloids: Dermatol; Iodopyrin; Benzosol; Agathin; Alumnol; Hypnal; Ipecac. sine Emetinâ; Tea. Emol-Keleet. Cocaine Spray.**—BURROUGHS, WELLCOME & Co., London.—We have previously called attention to the uses of Dermatol, Iodopyrin, Benzosol, and Agathin. These drugs are now offered to us in tabloid form, the convenience of which is so generally recognised. In addition to its value as an external application, Dermatol promises to be of some service in cases of gastric catarrh. In similar cases benefit may be anticipated from the administration of **Alumnol,** which is a sulphuric acid salt of aluminium, readily soluble in water, and highly astringent. **Hypnal** is a compound of chloral hydrate and antipyrine. **Ipecac. sine Emetinâ** should be a boon to those physicians who have to treat acute cases of dysentery. We have used it in a few cases, chiefly dysenteric diarrhœa and tubercular diseases of the lungs. In doses of thirty drops of the tincture, three or four times a day, it was distinctly beneficial, and never gave rise to any symptoms indicating an emetic action. Only Merck's preparation was employed, as several of those on the market contain a considerable percentage of emetine. We are glad to have the drug in tabloid. The **Tea** tabloids are so handy for the preparation of this refreshing beverage that they are likely to become very popular.

**Emol-Keleet** is a fine powder, closely resembling purified Fuller's earth to which it is however superior. It was highly spoken of by Dr. Allan Jamieson, of Edinburgh, at the Newcastle meeting of the British Medical Association. It has a very softening effect on epidermal masses sometimes met with in the hands and feet.

**The Cocaine Spray** is an ingenious little device, enabling a patient to dissolve a tabloid containing a known and limited quantity of cocaine and apply it to the nose, without producing the toxic effects of too large a dose. The apparatus is small enough to be carried in the waistcoat pocket, and is sold at a very cheap rate.

---

**Migranin. Loretin.**—FARBWERKE VORM. MEISTER LUCIUS & BRUENING, Hoechst o-Main, Germany.—We have received samples of these preparations from Messrs. Burroughs, Wellcome & Co., who are the agents for them in Great Britain and Ireland. **Migranin,** of which we have had no experience, is a new neurotic described by Dr. Martin Overlach as Citro-caffeine-antipyrin. He found that by its means not only were attacks of headache warded off, but it produced full remedial effects in the midst of one, while it had moreover a striking influence in diminishing the frequency of threatened attacks. It was found, after prolonged clinical experience, that the best dose of Migranin for adults is 17 grains dissolved in water, repeated if necessary at intervals of two hours.

**Loretin,** the chemical name of which is Iodo-oxy-quinoline-sulphonic acid, is an odourless antiseptic powder, similar to iodoform, and preferable to the latter on account of its freedom from odour and its non-poisonous qualities. We have tried it in one or two cases of chronic ulcer of the leg with great success. It is undoubtedly a strong antiseptic with no disagreeable qualities.

---

**Chirata Liquida, Kemp.**—THE LABORATORY, Kensington, London.—This fluid is a solution of definite strength in glycerine and water of the two bitter principles contained in our old friend Chiretta bark, two drachms being equal to half an ounce of the bark, or one pint of the infusion. If popularity be a test of utility, then the universal belief in the therapeutic value of this drug in India, gives the new purified and standardised solution a claim to our attention in England, where there is ample scope for the introduction of tonics superior to previously existing ones.

---

**Beef Peptone.**—Ferris & Co., Bristol.—This peptone has the following composition :

| | |
|---|---|
| Peptone albuminoids .. ... ... | 65.30 |
| Other Extractives ... ... ... ... ... | 3.10 |
| Water ... ... ... ... ... ... ... | 27.60 |
| Ash ... ... ... ... ... ... ... ... | 4.00 |
| | 100.00 |

Like all other peptones it has a nauseous bitter taste, which materially detracts from its utility as a food for the fastidious and sensitive invalid.

---

**Niemann's Syrup Carnis.**—A sample of this has been sent to us by Messrs. Ferris & Co., of Bristol, but there is no indication of its place of manufacture.

Raw meat juice in one form or another has become a necessity in the dietetics of the sick. As a rule the juice is preserved by chemical means ; but this preparation is free from drugs, and is made by simply mixing sugar with the juice expressed by hydraulic apparatus. It contains a large amount of soluble albumen, and has no disadvantages of taste or smell, to those who do not object to things sweet.

---

**Extract of Beef and Malt Wine.**—Wm. Glendenning and Sons, Newcastle-on-Tyne.—This combination of Alto-Douro Port, with Kepler Malt Extract, and Beef Jelly (Mosquera) containing 53.40 per cent. of albumen, forms a highly nutritious and palatable fluid, rich in diastase and peptone, needing no digestion, and therefore capable of speedy and easy absorption in all cases of chronic debility or exhaustion from acute disease.

---

**Syphons of Soda, Potash, Seltzer, and Lithia**—The Chemists' Aerated & Mineral Water Association, London.—This Association, which has established branches in several towns, of which Bristol is one, has sent us samples of the above, which are of excellent character. It is not generally known that the ordinary soda water of conviviality is a Pharmacopœia preparation, and as such should conform to the direction of official instruction. The Chemists' Association make a point of the definite strength of their waters, which should, therefore, specially commend themselves to medical men who require them for the sick room as well as for social purposes. B.P. also appears on their Potash and Lithia labels. Those who do not want their Soda and Potash Water of Pharmacopœia strength, can obtain these preparations with five grains to the half-pint instead of fifteen.

---

**Mexican Chocolate. Chocolate Biscuits. Ginger Chocolate. Pâté d'Abricot au Chocolat. Assorted Chocolates. Crême de la Duchesse. Bonbons Assortis. Coffee Crêmes.**—CADBURY BROS,, Bourneville, Birmingham.

In September, 1890, we gave a lengthy notice of Cadbury's Cocoa Essence. It is almost needless to say that this well maintains the reputation it has had for so long. In sending us some more of it, Messrs. Cadbury have accompanied it by many of the exquisite things for which the firm is renowned. They are excellent preparations for the sick person, who will find them invaluable for taking immediately after the nasty medicines which he often has to swallow. We have not been able to quite make up our mind as to which is the best of these delicious confections. We would, therefore, recommend that all of them should be procured and the purchaser decide for himself. If the stock should last over the illness, he will probably enjoy them quite as much when he is well or, if not, he will have no difficulty in getting his friends to help him out with them.

The consumption of cocoa is rapidly growing. It has an advantage over tea and coffee that should never be overlooked. It is not merely a conveyer of warmth into the system, nor does it only contain a powerful nitrogenous alkaloid; it is a true food; and in large quantities helps to build up the system and to supply it with a great deal of valuable fuel. At the annual meetings of the British Medical Association the cocoa exhibits are amongst the most interesting, and at Bristol this year we had opportunities of witnessing this. In our previous notice we cautioned our readers against those foreign cocoas which have a considerable addition of alkaline salts. It is far better to use articles of home manufacture which are prepared by firms of high standing.

---

**Stower's Lime Juice Cordial.**—ALEXANDER RIDDLE & CO., London.—This beverage was introduced in 1862, and has survived many a younger rival. The popularity of the lemon as the basis of a refreshing drink continually grows: lemon juice is no longer a disagreeable but necessary anti-scorbutic, the dose being administered daily under compulsion; but a lemon squash, or some substitute for it, has become a pleasure to be indulged in frequently. No better drink of the kind can be made than this cordial diluted with iced aerated water.

---

**Spt. Vini Meth. Lin. Aconiti, B.P. Lin. Belladonnæ. Lin. Camph. Co. Lin. Saponis Co. Ess. Limonis Nov. Opt.**—A. & J. WARREN, Bristol.—Samples of these preparations have been sent to us by Messrs. Warren, whose reputation as makers of methylated spirit is well known. The sample of spirit sent to us is unmineralised; that is, it is without the proportion of mineral naphtha which in 1891 the Government ordered to be added to all methylated spirit sold retail. Certain exceptions have since been granted by the authorities, and permission from the Inland Revenue to use methylated spirit in making Pharmacopœial liniments has of course had the effect of considerably cheapening them. All the **Liniments** we have received from Messrs. Warren have been prepared in this way. The **Essence of Lemon** is excellent, and is of value both in pharmacy and in cooking.

---

**Fels's Germicide Soap.**—FELS & Co., Philadelphia.—Messrs. Lorimer & Co. have forwarded us a sample of this. It contains small quantities of various antiseptics, including the naphthols, eucalyptol, methyl salicylate, and hydrarg. bichloride (1—2000), with sal alembroth (1—2000). Actual tests on culture fluids have shown that it has a direct antiseptic and sterilising power. For all cleansing purposes in connection with cases of scarlet fever, and for many other conditions where soap is of service, this particular variety must be of especial utility; but we do not admit the necessity for a combination of so many antiseptics, and it surely cannot be necessary to go all the way to Philadelphia for that which might easily be made on this side of the Atlantic.

---

**The "Riphokam."**—J. ROGERSON, London.—Proceeding on the theory that compared with the front part of the body the back is inadequately protected by the ordinary clothing, Mr. Rogerson has devised and patented a double-back under vest, which is well made, and which we can testify from personal experience is exceedingly comfortable to wear. The invention does not consist in adding to the thickness by merely laying one piece of material over another, but by the manner of weaving, the porous character of the garment is still preserved, while the risk of chill is lessened. If any of the obscure neuropathies or pulmonary troubles are caused by insufficient covering of the spinal column or back, this vest ought to be a prophylactic. Mr. Standerwick of Stokes Croft is Mr. Rogerson's local agent.

